# Inequality in prevalence, awareness, treatment, and control of hypertension in Iran: the analysis of national households’ data

**DOI:** 10.1186/s12889-022-14768-4

**Published:** 2022-12-14

**Authors:** Mahdi Mahdavi, Mahboubeh Parsaeian, Farshad Farzadfar, Efat Mohamadi, Alireza Olyaeemanesh, Amirhossein Takian

**Affiliations:** 1 National Institute of Health Research, Tehran University of Medical Sciences (TUMS), Tehran, Iran; 2 Bernard Lown Scholar Program in Cardiovascular Health, Harvard Chan School of Public Health, Boston, USA; 3 Department of Epidemiology and Biostatistics, School of Public Health, Tehran University of Medical Sciences (TUMS), Tehran, Iran; 4 Non-Communicable Diseases Research Center, Endocrinology and Metabolism Population Sciences Institute, Tehran University of Medical Sciences (TUMS), Tehran, Iran; 5 Health Equity Research Center (HERC), Tehran University of Medical Sciences (TUMS), Tehran, Iran; 6 National Center for Health Insurance Research, Health Insurance Organization, Tehran, Iran; 7 Department of Global Health and Public Policy, School of Public Health, Tehran University of Medical Sciences (TUMS), Poursina Avenue, Qods Street, Enqelab Square, Tehran, Iran; 8 Department of Health Management, Policy and Economics, School of Public Health, Tehran University of Medical Sciences (TUMS), Tehran, Iran

**Keywords:** Universal Health Coverage, Health Inequality, Hypertension, ‘Prevalence, Awareness, Treatment and Control’, Effective Coverage, Socioeconomic Status

## Abstract

**Background:**

Providing an equitable Universal Health Coverage (UHC) is key for progressing towards the sustainable development goals in the health systems. To help policymakers make hypertension services more equitable with existing (limited) resources in Iran, we examined the inequality of the prevalence, awareness, treatment, and control (PATC) of hypertension as the four indicators of hypertension UHC in Iran.

**Methods:**

This research was a cross-sectional study of inequality of PATC of hypertension using a representative sample of Iranians aged ≥ 25 years from the Iran 2016 STEP wise approach to Surveillance study (STEPS). Outcome variables consisted of PATC of hypertension. Covariates were demographic (age, sex, and marital status) and living standard (area of residence, wealth status, education, and health insurance) indicators. We drew concentration curves (CC) and estimated concentration indices (C). We also conducted normalized Erreygers decomposition analysis for binary outcomes to identify covariates that explain the wealth-related inequality in the outcomes. Analysis was conducted in STATA 14.1.

**Results:**

The normalized concentration index of hypertension prevalence and control was -0.066 (*p* < .001) and 0.082 (*p* < .001), respectively. The C of awareness and treatment showed nonsignificant difference between the richest and poorest. Inequality in the hypertension prevalence of females was significantly higher than males (C = -0.103 vs. male C = -0.023, *p* < .001). Our analyses explained 33% of variation in the C of hypertension prevalence and 99.7% of variation in the C of control. Education, wealth index, and complementary insurance explained most inequality in the prevalence. Area of residence, education, wealth status, and complementary insurance had the largest contribution to C of control by 30%, 28%, 26%, and 21%, respectively.

**Conclusions:**

This study showed a pro-rich inequality in the prevalence and control of hypertension in Iran. We call for expanding the coverage of complementary insurance to reduce inequality of hypertension prevalence and control as compared with other factors it can be manipulated in short run. We furthermore advocate for interventions to reduce the inequality of hypertension control between rural and urban areas.

**Supplementary Information:**

The online version contains supplementary material available at 10.1186/s12889-022-14768-4.

## Introduction

Hypertension (HTN) is a leading noncommunicable disease affecting health and wellbeing of a large population of the world. It is a major risk factor of cardiovascular diseases globally [1]. In a rising trend, the prevalence of HTN will mount to 29% by 2025 globally [2]. The prevalence of HTN in the region of the Middle East and North Africa was 26.3% in 2014 among adults aged ≥ 18 years. Of Iranians aged ≥ 25 years, 30% were living with HTN in 2016 [2].

Universal Health Coverage (UHC) is a key tracer for monitoring countries’ achievements in sustainable development goals [3]. It is at the core of the “Sixth Five-Year Economic, Social, and Cultural Development Plan” of Iran too [4]. UHC prescribes that all individuals should receive health services they need without falling into poverty and services are of high quality. UHC is measured through four indicators consisting of prevalence, awareness, treatment, and control of health condition [5]. A country’s progress towards UHC is not only measured in terms of the level of UHC, but also in terms of equitable distribution of the UHC indicators among socioeconomic groups. Without adequate measurement of inequality, disadvantage populations will continue to be underutilized or receive no treatment at all and consequently remain uncontrolled. As a result, a country may enhance UHC indicators in a long run, yet improvements may remain pro-rich. In this research, we therefore examined whether and to what extent the distribution of UHC indicators prevalence, awareness, treatment, and control of hypertension systematically vary by socioeconomic status and which socioeconomic factors explain the distribution of the outcomes.

Inequality manifests a disparity in the health outcome that can be explained by the socioeconomic factors. It represents the degree of association between rates for a health indicator and the distribution of population among ordered groups based on socioeconomic status (SES) [6]. O’Donnell and colleagues used term ‘living standard’ to denote SES and provide direct, e.g., income and consumption, and indirect proxies, e.g., wealth index developed through a principal component analysis or factor analysis, to measure living standard [7]. Education, occupational status, neighborhood, and health insurance have also been used as SES proxies. At household level, SES includes demographic data such as place of residence to account for rural/urban socioeconomic differences [8, 9]. However, the choice of SES measures is not straightforward as it depends on various factors [8]. Each proxy displays different relationships with various health outcomes and would be addressed by different policies [10].

**Fig. 1:**
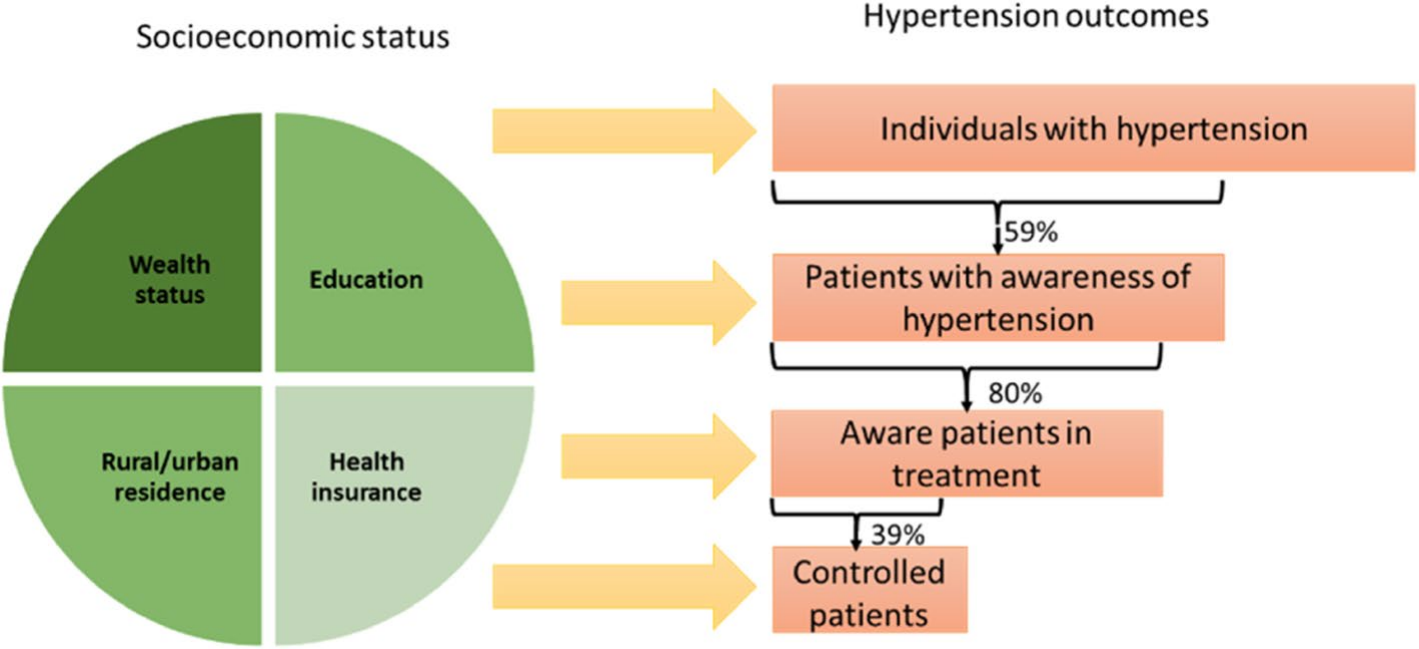
Socioeconomic status and prevalence, awareness, treatment, and control of hypertension Note: Hypertension outcomes are measured in a stepwise approach; of individual with hypertension, 59% are aware that have hypertension. Of those with awareness, 80% are in-treatment and of those treated, 39% are under control [2]

Hypertension has multiple dimensions that are relevant for examining universal health coverage. Hypertension outcomes are among the most common indicators for measuring health inequality in the world. Hypertension coupled with “life expectancy”, “infant mortality”, “obesity and overweight (body mass index)” and “mortality rate” have been most common indicators of health inequity [11]. As shown in Fig. 1, a direct link can be made between hypertension outcomes and SES.

Inequality driven by income, education, employment, and residence area is vastly reported in the literature. Strong evidence from 283 studies shows that low income, low SES, or low level of education are positively associated with the presence of noncommunicable diseases [12]. Income made the greatest contribution to inequality of late-life health [13]. The inequality of hypertension between poor and rich is increasing in the United States and the United Kingdom [14]. In Iran, a significant socioeconomic inequality in hypertension prevalence was reported disfavoring the poorest group and the distribution of hypertension at the disadvantage of women [15]. However, there is a paucity of evidence regarding the magnitude, direction, and contributing factors of inequality of awareness, treatment, and control in Iran. We measured inequality in the prevalence, awareness, treatment, and control of hypertension in Iran. Aiming to foster progress towards universal coverage of hypertension services, this study aspires to provide evidence for health system improvement in Iran. Our findings are expected to help policymakers take initiatives to improve health system for making hypertension services equitable with the existing (limited) resources.

## Methods

This research was a cross-sectional study of inequality of PATC in Iran. The study population consisted of a representative sample of Iranians aged ≥ 25 years from urban and rural areas (27,738 participants) across the country in 2016.

### Outcomes

Outcome variables consisted of prevalence, awareness, treatment, and control of hypertension. Prevalence measures the effectiveness of preventive services in reducing disease events. Prevalence refers to rate of individuals with blood pressure with systolic blood pressure (SBP) ≥ 140 mmHg or diastolic blood pressure (DBP) ≥ 90 mmHg based on the evidence-based recommendations for treatment and management of high blood pressure proposed by the Eighth Joint National Committee (JNC8) in 2014. Furthermore, the self-reported use of antihypertensive can also be considered as the presence of HTN [16]. Awareness refers to if a patient knows about his/her hypertension. Awareness was deemed to be present if an individual answered ‘Yes’ to the question ‘Have you ever been diagnosed with hypertension by a physician or a health professional?’ Treatment refers to pharmacological therapy of hypertension during two weeks prior to the study. Hypertension control/controlled hypertension refers to an average SBP < 140 & DBP < 90 mmHg based on the JNC8 [17].

### Explanatory factors

Explanatory factors comprised of demographic and living standard factors. Demographic factors consisted of age, sex, and marital status (Table 1). Living standard was measured through the area of residence (rural/urban dwelling), wealth index, education, and health insurance coverage. Wealth index represents a composite measure of cumulative living standard of a household, which was constructed using principal component analysis [18]. It was estimated using data from ownership of certain assets (e.g. house, car), home appliances (e.g. fridge, washing machine) and household facilities (e.g. the type of access to water) [19, 20]. Basic insurance coverage refers to being under coverage of a basic health insurance. Complementary health insurance is a coverage policy that reimburse the surcharges of medical services not being reimbursed by basic health insurance or services delivered by private providers [21].

**Table 1:** Covariates of inequality analysis of hypertension prevalence, awareness, treatment, and control

Variable	Description
Prevalence	Systolic blood pressure (SBP) ≥ 140 mmHg or diastolic blood pressure (DBP) ≥ 90 mmHg based on JNC8 or the self-reported use of antihypertensive medications [17]
Awareness	Awareness is present if an individual answered ‘Yes’ to the question ‘Have you ever been diagnosed with hypertension by a physician or a health professional?’
Treatment	Pharmacological therapy of hypertension during two weeks prior to the study [16]
Control	An average SBP < 140 & DBP < 90 mmHg based on the JNC8 [17]
Age	Grouped as 25–34, 35–44, 45–54, 55–64, 65–74, and 75 + years
Sex	Female or males
Marital status	Two groups: married or single/divorced/separated
Area of residence	Two groups: rural residents or urban residents
Wealth status	Grouped into five quintiles ranging from the poorest to richest
Education	Categorized into four groups: participants with no schooling, with 1–6 years of schooling, with 7–12 years of schooling, or higher than 12 years of schooling
Basic insurance coverage	Having a basic insurance coverage or no basic insurance coverage
Complementary health insurance	Having a complementary insurance coverage or no complementary insurance coverage

### Data

We used the data that were collected through Iran 2016 STEPS study. The STEPS study was conducted based on the WHO STEPwise approach to Surveillance (STEPS) in Iran. The STEPS 2016 included a representative sample of Iranian urban and rural dwellers across 30 provinces (27,738 participants), which were selected based on a multistage random sampling method. The methods employed in the Iran STEPS 2016 study including the sampling design, the validity and reliability of the study questionnaire, the interview guide, and data collection methods were presented elsewhere [22]. The variables used in this research were all self-reported except for blood pressure that was measured through trained personnel. It was measured on the right upper-arm three times, having had the participant rested for 5 min in a seated position [23]. An average of the last two measurements was considered as the blood pressure measure.

### Analyses of inequity

We conducted analyses to draw concentration curve (CC), to estimate concentration index (C), and to identify explanatory variables.

### Concentration curve

Concentration curves determined whether inequity in the outcomes exist, and which wealth group is favored. It shows the cumulative percentage of individuals with prevalence, awareness, treatment, or control of hypertension (y-axis) in relation to a cumulative percentage of individuals ranked by the wealth index from the lowest to highest wealth index (x-axis). In case there is no health inequality related to wealth, everybody would have the same value of health outcomes, and CC would be 45-degree line that runs from the bottom left-hand to the top right-hand corner. This line is called the line of equality. The higher rate of prevalence among poorer compared to wealthier people would be displayed by CC above the line of inequality. By contrast, CC of awareness, treatment, and control below the line of equality would be at disadvantage of poorest group. We depicted CC using the command glcurve and two-way command in STATA 14.1.

### Concentration index

This analysis measured the magnitude of inequality that was used for comparison between groups. It was defined as twice the area between CC and the line of equality. O’Donnell and colleagues introduced a convenient formula for computation of concentration index, that defines it in terms of the covariance between the health variables and the fractional rank in the living standard distribution.

1 $$C=\frac{2}{\mu }cov (h,r)$$

In this study, $h$ was the hypertension outcome, $\upmu$ was its mean, and $r$ was the fractional rank of individual in wealth index from the poorest to the richest. The range of the concentration index is from − 1 to 1. A negative concentration index for prevalence (ill health), implied disproportionate concentration of hypertension among poor. In contrast, positive values of concentration index for awareness, treatment, and control of hypertension (good health) indicated poorer people are disadvantaged. In case of unbounded outcomes, the concentration index tends to lie between -1 and 1. However, for outcomes that are binary, alike outcomes examined in this research, the feasible interval of the index is not –1 and 1. As noted by Wagstaff, in this case with increase in the mean of an outcome, its concentration index shrinks. For binary outcomes, the concentration index for large samples is between μ − 1 for the lower bound and 1 − μ for the upper bound. Therefore, Wagstaff proposed to normalize the concentration index by dividing through by $1-\mu$. The formula for estimating normalized concentration index can be written as below [24].

2 $$C=\frac{2}{n\mu }\sum_{i=1}^{n}{h}_{i}{r}_{i}-1$$

where $n$ is the sample size. The rest of notations is the same as Eq. 1.

## Decomposition analysis

In the previous step, C was used to determine the magnitude of inequality in hypertension outcomes. We took a further step to explain inequality by using a decomposition method. The analysis of decomposition relied on explaining distribution of hypertension outcomes by the explanatory factors that may systematically vary with wealth status. The decomposition method demonstrated the contribution of the determinants of wealth to health inequality. The contribution is a product of the sensitivity of hypertension outcomes with respect to an explanatory factor (elasticity) and the degree of wealth-related inequality in that factor (concentration index). We decomposed inequality by age, sex, marital status, area of residence, education, basic health insurance, complementary health insurance, and wealth index to determine contributing factors of PATC of hypertension [7]. Each hypertension outcome ($y$) can be described as a linear regression model as below:

3 $$y=\alpha +{\sum }_{k}{\beta }_{k}{x}_{k}+\varepsilon (1)$$

where ${x}_{k}$ is a regressor i.e., explanatory factor and ${\beta }_{k}$ is the coefficient of that regressor. Then the concentration index for $y$ can be written as follows:

4 $$C={\sum }_{k}({\beta }_{k}{\overline{x}}_{k}/\mu ){C}_{k}+G{C}_{\varepsilon }/\mu$$

where $\mu$ is the mean of $y$, $\overline{x}$ is the mean of ${x}_{k}$, ${C}_{k}$ is the concentration index of ${x}_{k}$, and $G{C}_{\varepsilon }$ is the generalized concentration index of the error term ($\varepsilon$). Eq. 4 shows that $C$ is equal to a weighted sum of the concentration indices of the $k$ regressors, where the weight for ${x}_{k}$ is the elasticity of $y$ with respect to ${x}_{k}\left({\mu }_{k}={\beta }_{k} \frac{{\overline{x} }_{k}}{\mu }\right)$. The residual component, the last term in equations showed the socioeconomic inequality in hypertension outcomes that was not explained by systematic variation in the regressors by socioeconomic status. In a well-specified model this should approach zero. For a bounded binary outcome like the hypertension outcomes, $C$ can be normalized through Eq. 5 as [25]:

5 $$C=\frac{C}{1-\mu }=\frac{{\sum }_{k}({\beta }_{k}{\overline{x}}_{k}/\mu ){C}_{k}}{1-\mu }+\frac{G{C}_{\varepsilon }/\mu }{1-\mu }$$

For interpreting results of decomposition analysis, we reported three pieces of information; the elasticities of the hypertension outcomes with respect to covariates, concentration index for each covariate, and total contribution of each covariate to the outcome’s concentration index. Above information derives from Eq. 5. Total contribution of each covariate was a product of elasticity of outcome with respect to a covariate and a concentration index attributed to that covariate. Residual term determined the unexplained variation in socioeconomic-related inequality in the hypertension outcomes.

We tested three statistical models: generalized linear models (GLM), probit models, and logit models (Eq. 3). GLMs refer to models with a response variable that follows exponential family distribution and is a nonlinear function of the covariates. To test if variable selection (specifying covariates for a regression model) was appropriate, we used Linktest. This test if passed, i.e., non-significant, shows that covariates were adequate to explain the outcomes. This test revealed if covariates are misspecified [26].

All analysis was conducted in STATA version 14.1. We used codes and analysis packages developed by O’Donnell et al. that are made publicly available by the World Bank [7].

## Results

After excluding 573 (2%) out of 27,738 participants, we considered 27,165 participants for analysis, of whom about 70% were between 25 and 54 years old. We found that the prevalence of hypertension was 30% (95% confidence interval (CI): 29.2–30.6). Fifty-nine percent (58.0–60.3) of hypertensive individuals were aware and 80% (78.9–81.4) of hypertensive & aware individuals were receiving treatment. The control rate of HTN was 39% (37.4–40.7).

### Concentration index and concentrative curve of hypertension outcomes

The normalized concentration index of hypertension prevalence, awareness, treatment, and control was -0.066 (*p* < 0.001), -0.017 (*p* = 0.193), -0.014 (*p* = 0.291), and 0.082 (*p* < 0.001), respectively (Table 2). There was significant inequality in the prevalence and control of hypertension between rich and poor.

**Table 2:** Concentration analysis of hypertension prevalence, awareness, treatment, and control of hypertension at the country level

**Outcomes**	**No of observations**	**Concentration index value**	**Standard error**	***P* value**^a^	**F test**	***P* value**^b^
Hypertension prevalence						
Female	13,745	-0.103	0.009	0.000	36.268	0.000
Male	12,960	-0.023	0.009	0.014		
Total	26,435	-0.066	0.007	0.000		
Awareness						
Female	4,398	-0.018	0.017	0.276	0.578	0.447
Male	3,583	0.024	0.020	0.230		
Total	7981	-0.017	0.013	0.194		
Treatment						
Female	2940	-0.011	0.017	0.531	0.296	0.587
Male	1805	-0.010	0.022	0.651		
Total	4745	-0.014	0.013	0.291		
Control						
Female	2386	0.090	0.023	0.000	0.373	0.542
Male	1400	0.059	0.032	0.061		
Total	3786	0.082	0.019	0.000		

^a^Within-group analysis of inequality

^b^Comparison of inequality between men and women

**Fig. 2:**
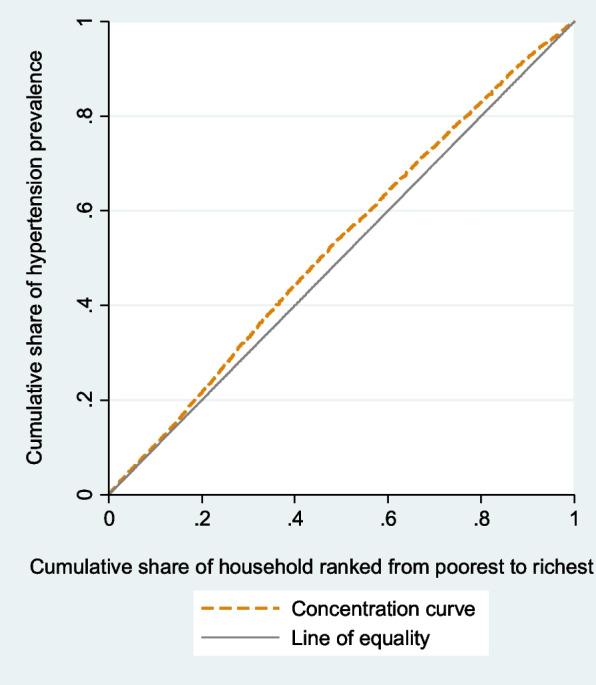
Concentration curve of hypertension

**Fig. 3:**
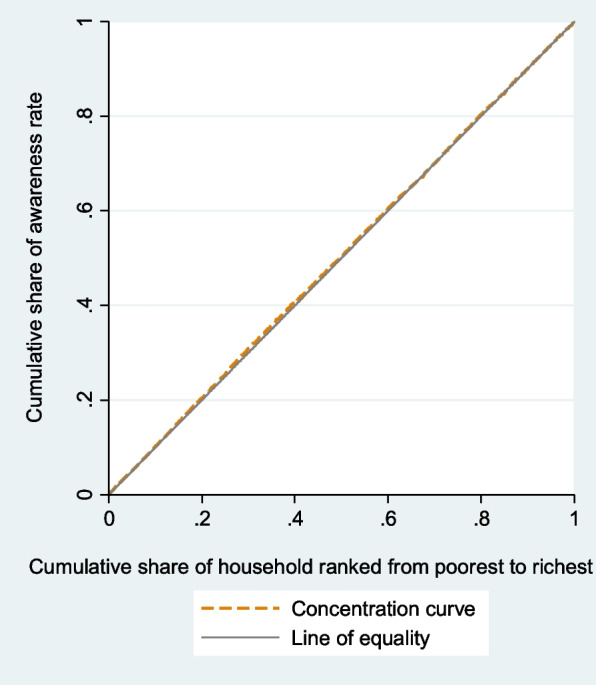
Concentration curve of awareness

**Fig. 4:**
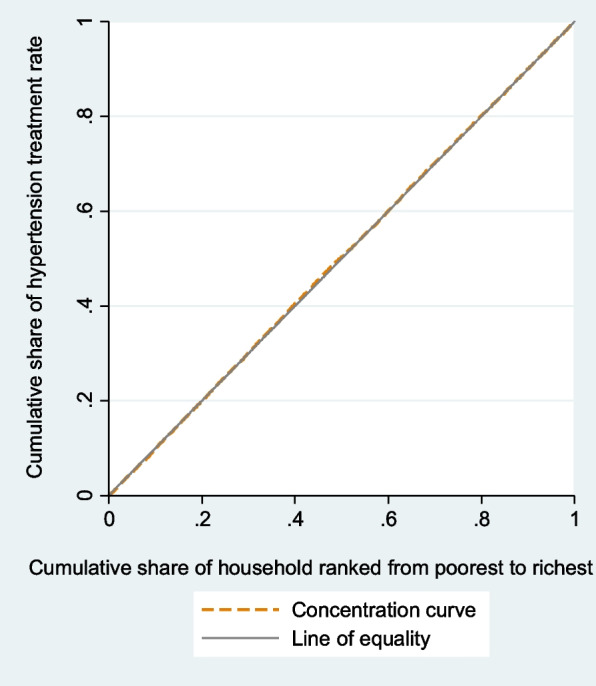
Concentration curve of treatment

The negative C of hypertension prevalence and its CC (Fig. 2) showed that the prevalence of hypertension was concentrated among poor people. CC of hypertension prevalence displays that cumulative share of hypertension prevalence rate decreases with the cumulative share of wealth index. The CC of prevalence lies above the line of equality, indicating that the prevalence of hypertension is concentrated more on worst-off. Concentration analysis of awareness and treatment shows nonsignificant difference between rich and poor. The CC for awareness (Fig. 3) and treatment (Fig. 4) lie on the line equality, indicating that inequality of awareness and treatment is close to zero in Iran.

**Fig. 5:**
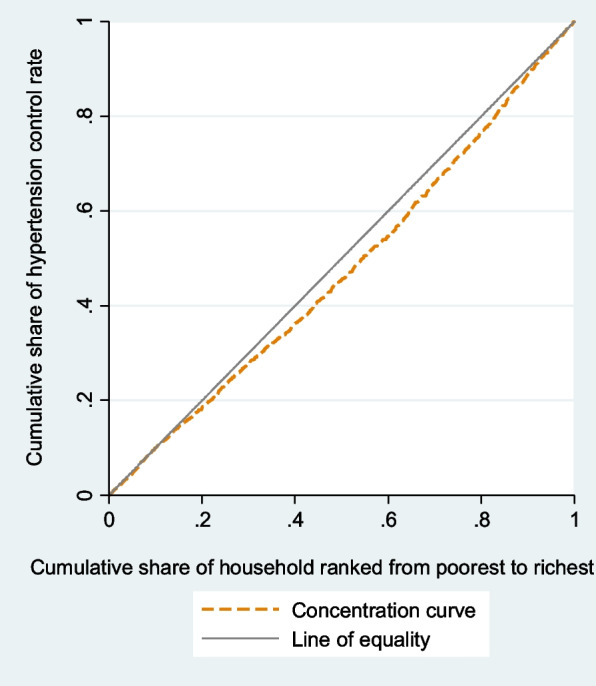
Concentration curve of control

Since control is a good outcome, the positive concentration index, in contrast to hypertension prevalence that is an ill health, indicated the concentration of control among the richest group. The concentration curve for the control of hypertension lies below the line of equality (Fig. 5).

Our results show that the inequality in the hypertension prevalence of women was statistically higher than men (female C = -0.103 vs. male C = -0.023, *p* < 0.001). Assuming equal variance and large sample, null hypothesis, i.e., no difference between wealth-related inequality of hypertension prevalence between female and male, was rejected (F test = 36.268, *p* < 0.001 and Z test = 6.12, *p* < 0.001). Regarding within-group difference, we found that there was wealth-related inequality of prevalence among female and among men (see *p* value* in Table 2).

Despite this, we did not find significant wealth-related inequality of awareness, treatment, and control between women and men (see *p* value ** in Table 2). The wealth-related inequality of control within female group was statistically significant (C = 0.090, *p* < 0.001).

The null hypothesis that there is no difference between provinces regarding C of prevalence was strongly reject (*F* = 3.829, *p* < 001). Out of 30 provinces, 14 provinces had statistically significant inequalities of hypertension prevalence at the disadvantage of the poorest groups. The differences in the inequalities of awareness and treatment of hypertension were present at the provincial level, (*F* = 1.610, *p* < 0.05 and *F* = 1.58, *p* < 0.05). At this level, most inequality was at the disadvantage of poorest groups though there were also provinces where inequality was at the disadvantage of richest group e.g., Tehran. The overall difference between the provinces in terms of hypertension control was nonsignificant (*F* = 1.292, *p* = 0.136).

#### Decomposition analyses

The decomposition analysis explained 33% of variation in the C of hypertension prevalence. The Linktest comparing between the GLM model and two logistic regression models showed superior performance of the GLM model (coefficient _hatsq = 0.025, *p* = 0.07) (Table 3).

**Table 3:** Decomposing socioeconomic inequality of hypertension prevalence

Categories	Coefficient	Elasticity	Concentration index (C)	Absolute contribution to C	Relative contribution to C per category of variable (%)	Relative contribution to C per variable (%)
Age group						-8.9
25–34	1					
35–44	0.145	0.136	0.032	0.004	-6.5	
45–54	0.289	0.229	0.056	0.013	-19.5	
55–64	0.395	0.248	0.005	0.001	-1.8	
65–74	0.481	0.170	-0.034	-0.006	8.7	
≥ 75	0.507	0.116	-0.058	-0.007	10.2	
Gender						2.7
Female	1					
Male	-0.025	-0.049	0.036	-0.002	2.7	
Marital status						2.3
Single/divorced	1					
Married	0.028	0.021	-0.072	-0.002	2.3	
Area						
Rural	1					0.3
Urban	0.000	0.000	0.477	0.000	0.3	
Basic insurance						0.2
No	1					
Yes	-0.007	-0.026	0.005	0.000	0.2	
Complementary insurance						-7.4
No	1					
Yes	0.020	0.018	0.271	0.005	-7.4	
Years of schooling						34.8
No schooling	1					
1–6 years	-0.032	-0.034	-0.135	0.005	-7.1	
7–12 years	-0.061	-0.093	0.167	-0.016	23.7	
> 12 years	-0.076	-0.056	0.213	-0.012	18.1	
Wealth status						8.8
Poorest	1					
Poor	0.009	0.007	-0.311	-0.002	3.5	
Average	0.009	0.007	-0.024	0.000	0.3	
Rich	0.004	0.003	0.309	0.001	-1.6	
Richest	-0.009	-0.007	0.602	-0.004	6.7	
C explained				-0.022	32.8	
Residual				-0.044		
C				-0.066		

The C of covariates was responsible for the large contribution of education and wealth status to C of prevalence. The negative C in the last row showed that hypertension prevalence was concentrated among them (C = -0.311 and -0.024 respectively). Decomposition analysis of prevalence showed that education, wealth status, and complementary insurance explained a large share of explained variation in the C of prevalence. Education and wealth status explained 35% and 9% of inequality in prevalence. The large concentration indices of wealth groups and education were responsible for greater contribution of these covariates for explaining inequality of prevalence. The contribution of age to C of prevalence was explained by the larger elasticities of age groups to hypertension prevalence. This was indicative of sensitivity of prevalence to individual age.

Our analyses explained 99.7% of variation in the C of hypertension control (Table 4). Consequently, the value of residual was close to zero. The result of linktest was non-significant (_hatsq coefficient = -0.058, *p* = 0.84) when we analyzed the decomposition of hypertension control using the GLM model. Area of residence, education, wealth status, and complementary insurance had the largest contribution to C by 30%, 28%, 26%, and 21%, respectively. The positive concentration indices show that there was inequality in hypertension control at the advantage of the poorest group. The large concentration indices of these variables (e.g., being urban resident C = 0.473) were responsible for their large contribution to C of hypertension control.

**Table 4:** Decomposing socioeconomic inequality of hypertension control in Iran

Categories	Coefficient	Elasticity	Concentration index (C)	Absolute contribution to C	Relative contribution to C per category of variable	Relative contribution to C per variable
Age group						-5.8
25–34	1					
35–44	-0.115	-0.027	0.019	0.000	-0.6	
45–54	-0.223	-0.168	0.087	-0.015	-17.7	
55–64	-0.225	-0.273	0.065	-0.018	-21.4	
65–74	-0.251	-0.250	-0.040	0.010	12.0	
≥ 75	-186	-0.140	-0.129	0.018	21.9	
Gender						1.0
Female	1					
Male	0.005	0.008	0.105	0.001	1.0	
Marital status						
Single/divorced	1					-0.8
Married	0.005	0.005	-0.130	-0.001	-0.8	
Area						29.7
Rural	1					
Urban	0.018	0.052	0.473	0.024	29.7	
Basic health insurance						
No	1					0.2
Yes	0.003	0.011	0.015	0.000	0.2	
Complementary insurance						21.2
No	1					
Yes	0.037	0.048	0.365	0.017	21.2	
Years of schooling						28.2
No schooling	1					
1–6 years	0.069	0.083	0.068	0.006	6.8	
7–12 years	0.070	0.058	0.269	0.016	18.9	
> 12 years	0.061	0.024	0.085	0.002	2.5	
Wealth status						25.9
Poorest	1					
Poor	-0.009	-0.008	-0.293	0.002	2.8	
Average	-0.001	-0.001	0.040	0.000	-0.1	
Rich	0.015	0.012	0.350	0.004	4.9	
Richest	0.043	0.029	0.523	0.015	18.2	
C explained				0.0821	99.7	
Residual				0.0002	0.3	
C				0.0823		

The contributions of age groups to concentration index of hypertension control were small as age groups cancel out each other’s contribution to the outcome. Furthermore, the age groups had smaller concentration index and greater elasticity. The large elasticity of age groups explained most of their contributions to the C of control. The elasticities of age groups are higher than the elasticities of wealth or education groups.

## Discussion

Using cross sectional data from the Iran 2016 STEPS study, this research aimed at providing a comprehensive analysis of wealth-related inequality of the UHC indicators for patients with hypertension in Iran. At the country level analysis, hypertension prevalence and control were slightly distributed at the disadvantage of poorest groups. Our study confirmed the minor pro-rich inequality of hypertension prevalence in Iran [15]. Studies report the pro-rich inequality of hypertension prevalence in the low-and middle-income countries such as Kenya [27] and China [28]. Yet, the magnitude of wealth-related inequalities in hypertension tends to be higher in poorer than in richer countries. In the lower income countries, hypertension awareness and treatment rates were observed to be higher among well-off households, while a hypertension control rate tended to be pro-rich regardless of the economic development level of those countries [29].

In a head-to-dead comparison between female and male, we found that the extent of inequity in the hypertension prevalence of women was statistically higher than the extent of inequality among men. One previous study also reported unequal distribution of hypertension at the disadvantage of women in Iran [15].

We did not find significant difference in the wealth-related inequality of awareness, treatment, and control between females and males. Regarding within-group difference, we found that there was wealth-related inequity of prevalence among females as well as among males. Furthermore, wealth-related inequity of control was only observed within female group (C = 0.090, *p* < 0.001). The concentration indices of hypertension prevalence, awareness, and treatment were significantly different between provinces. Despite this, control was not significantly unequal between provinces. The statistically significant inequity of prevalence was observed in 14 out of 30 provinces.

Decomposition analysis of prevalence showed that education, wealth status, and complementary insurance explained a large share of explained variation in the C of prevalence. Education and wealth status explained 35% and 9% of inequality in prevalence. In a study conducted in Kenya, about 10% of inequity in hypertension prevalence was explained by wealth index, 9% by education and 7% by paid employment. Sociodemographic factors explained 18% of inequality [27]. In total, our model explained 33% of variation in C of hypertension prevalence, in contrast, Gatimu et al. explained 99.7% of inequality in hypertension. However, they included body mass index that explained 47% of variation in the inequality of hypertension.

Most of the pro-rich inequality in hypertension control in Iran was explained by living in the urban area, complementary insurance, wealth index, and education, which confirms the established associations between health state and wealth status and education [27, 30, 31]. The underlying argument that explains such association is that wealth or income is materialized in resources such as nutrition and housing that affects health and wellbeing [32].

The main socioeconomic factors that explain most inequality in hypertension prevalence and control consist of the education, wealth status, and complementary insurance. Among these factors, basic health insurance and complementary insurance are of greater policy relevance as being related to the depth of coverage and degree of risk protection by insurance schemes. Almost the entire population of Iran has access to a basic insurance coverage, this explains why the basic insurance made no contribution to the explained variation in hypertension control. In a sharp contrast, complementary insurance made significant contributions to explaining variation in inequality of hypertension prevalence and control. We therefore call for extending complementary insurance coverage, as a means to extend the coverage of services, to reduce the inequality of hypertension prevalence and control in the country.

Hypertension treatment is a measure of crude coverage. This indicator was measured with a single question assessing if patient takes antihypertension medications. While it is an indicator of general access to hypertension medication, it does not measure if patients receive right treatment or doses. In this case, the inequality in treatment could have been different and factors like area of residence may affect it differently.

Despite hypertension treatment, the control of hypertension is an indicator of much complex outcome and goes far enough to embrace the quality of care as translated to improved access to medical care, continued utilization of services, and accessibility of multiple treatment lines for hypertension, and multimorbidity management. Living in urban areas is much likely to provide quality care and to improve hypertension control given the availability of advanced medical technologies and exhaustive list of medical specialists and care facilities that would improve the likelihood of hypertension control for patients living in these areas. As a result, the area of residence made a significant contribution to inequality of hypertension control. In contrast, the area of residence made no contribution to hypertension prevalence as the prevalence is dependent on healthy lifestyle that can be kept everywhere.

We showed that there was no wealth-related inequality in the awareness of hypertension between the poorest and richest group of Iranian population. However, this finding should be interpreted with caution as the question to measure awareness is prone to a measurement limitation. Multiple questions to measure awareness of hypertension are required to get an accurate picture of patient awareness of hypertension. Some other countries break down awareness to the knowledge of individuals about the impact of hypertension on heart, vessels, and body organs and awareness of healthy lifestyle for hypertension. For example, in Canada surveys with multiple questions have been used to measure each of these conditions [33]. With a such measurement, analysis may show different picture regarding the distribution of outcome between the poorest and richest groups.

This research faced the challenge of causal inferences from cross-sectional survey data. Causal relationship is paramount for developing valid evidence for policymakers to understand what factors need to be considered for reducing inequality. While we claim no causal relationship, we draw attention to some criteria that help maintain causal relationships; we relied on a compelling theoretical model with regard to examining preceding factors for the prevalence and control of hypertension [34].

We considered wealth index as the main living standard measure. However, a key question is what happens to inequality when living standard varies. Since we have no access to income data, we were unable to conduct the sensitivity analysis that tests living standard over income while other variables were held constant [35]. One province did not participate in the STEPS study. Still, we believe that the external validity of our findings is reasonably maintained by the multistage random sampling methods with proportional to size samples. Given this, the validity of inferences might be maintained over variations in persons or times [36].

While the regression model that was used for decomposing inequity in hypertension prevalence passed a Linktest (that examines specification of variables to explain the outcome), the specification of variables can be markedly improved. This residual tends to be close to zero in well-specified models. Other studies have involved lifestyle behaviors to improve the model fit [27], though it might be irrelevant for analysis of inequality as our interest was to measure effects of SES on the health outcomes rather than the effect of behaviors that derive from SES.

## Conclusions

This study showed a pro-rich inequality in the prevalence and control of hypertension in Iran. We did not however find significant inequality in the awareness and treatment of hypertension in Iran. We found pro-rich inequality between men and women and between provinces. However, inequality at disadvantage of better-off was also found, for example, inequality in treatment that disfavors richest groups in some provinces such as Tehran. The main socioeconomic factors that explained most inequality in hypertension prevalence and control consist of the education, wealth status, and complementary insurance. Among these factors, basic health insurance and complementary insurance are of greater policy relevance as being related to the depth of service coverage and financial risk protection. Almost the entire population has access to a basic insurance coverage, subsequently this factor has made no contribution to explained variation in hypertension control which is in sharp contrast with pro-rich complementary insurance. We call for expanding complementary insurance coverage to reduce inequality of hypertension prevalence and control in the country. We also call for improved health system’s operations to reduce pro-urban inequality of hypertension control.

## Supplementary Information

**Additional file 1. **

## Data Availability

The datasets analysed in the present study are not publicly available due to strict rules and regulations but are available from the corresponding author on a reasonable request.

## Abbreviations

UHC: Universal Health Coverage
HTN: Hypertension
PATC: Prevalence, awareness, treatment, and control
SES: Socioeconomic status
SBP: Systolic blood pressure
DBP: Diastolic blood pressure
STEPS: WHO STEPwise approach to Surveillance
CC: Concentration curve
C: Concentration index
