# Supplementary material for: Inequality in prevalence, awareness, treatment, and control of hypertension in Iran: the analysis of national households’ data
Source: BMC Public Health. 2022 Dec 14;22:2349. doi: 10.1186/s12889-022-14768-4 (PMC9753315; doi:10.1186/s12889-022-14768-4)
Supplement: Supplementary file 1 — Additional file 1. [file 12889_2022_14768_MOESM1_ESM.docx]

The prevalence of hypertension based on the socioeconomic characteristics of study participants that were considered for the analysis is given in Table 1.

Table 1. Prevalence of hypertension based on socioeconomic characteristics of study participants

|  | JNC8 | |
| --- | --- | --- |
|  | %^†^ | 95% CI |
| Overall | 29.9 | 29.2-30.6 |
| Age groups (years old) |  |  |
| 25-34 | 7.3 | 6.7-8.0 |
| 35-44 | 16.3 | 15.4-17.4 |
| 45-54 | 33.1 | 31.8-34.5 |
| 55-64 | 50.6 | 49.0-52.2 |
| 65-74 | 64.0 | 62.1-66.0 |
| ≥75 | 69.7 | 67.3-72.1 |
| Gender |  |  |
| Male | 27.9 | 27.1-28.8 |
| Female | 31.7 | 30.9-32.5 |
| Area of residence |  |  |
| Urban | 29.4 | 28.6-30.2 |
| Rural | 31.1 | 29.9-32.4 |
| Wealth status |  |  |
| Poorest | 31.8 | 30.3-33.3 |
| Poor | 35.0 | 33.5-36.5 |
| Average | 30.8 | 29.4-32.2 |
| Rich | 27.9 | 26.5-29.3 |
| Richest | 25.2 | 23.9-26.5 |
| Years of schooling |  |  |
| No schooling | 56.4 | 54.8-58.0 |
| 1-6 years | 34.4 | 33.2-35.7 |
| 7-12 years | 20.6 | 19.7-21.5 |
| >12years | 19.8 | 18.6-21.1 |
| Complementary health insurance coverage |  |  |
| No | 27.8 | 27.1-28.5 |
| Yes | 37.2 | 35.8-38.6 |

We found that the prevalence of hypertension was 29.9% (95% confidence interval (CI): 29.2-30.6) (Table 1). The prevalence rate sharply increased by age from younger to older groups, reaching its peak at 69.7% among those ≥75 years old. The prevalence percentage was lower among males and urban dwellers. Among the wealth groups, the richest group had the lowest prevalence. The prevalence also decreased from 56.4% among illiterate group to 19.8% among those with >12 years of schooling.

The awareness of hypertension based on the socioeconomic characteristics of study participants is given in Table 2. We found that 59.2% (58.0-60.3) of hypertensive individuals were aware (Table 2). Awareness markedly increased by age, from 21.3% in the youngest to 76.8% in the oldest group. Awareness rate of females was higher than males. Awareness declined with increases in years of schooling, reaching its lowest among the well-educated group. Patients with a higher awareness were more likely to have complementary insurance coverage.

Table 2. Percentage of hypertension awareness based on socioeconomic characteristics of study participants

|  | JNC8 | |
| --- | --- | --- |
|  | % ^†^ | 95% CI |
| Overall | 59.2 | 58.0-60.3 |
| Age groups (years old) |  |  |
| 25-34 | 21.3 | 17.9-25.1 |
| 35-44 | 34.7 | 31.5-37.9 |
| 45-54 | 54.2 | 51.7-56.6 |
| 55-64 | 66.9 | 64.8-68.9 |
| 65-74 | 71.7 | 69.3-74.0 |
| ≥75 | 76.8 | 74.0-79.4 |
| Gender |  |  |
| Male | 49.9 | 48.2-51.7 |
| Female | 66.7 | 65.2-68.1 |
| Area of residence |  |  |
| Urban | 59.5 | 58.0-60.9 |
| Rural | 58.5 | 56.3-60.6 |
| Wealth status |  |  |
| Poorest | 60.2 | 57.5-62.8 |
| Poor | 60.4 | 58.0-62.7 |
| Average | 58.2 | 55.6-60.8 |
| Rich | 58.2 | 55.5-60.9 |
| Richest | 58.8 | 55.8-61.7 |
| Years of schooling |  |  |
| No schooling | 70.8 | 68.8-72.7 |
| 1-6 years | 59.0 | 56.9-61.2 |
| 7-12 years | 49.6 | 47.3-51.9 |
| >12 years | 51.2 | 47.8-54.5 |
| Complementary health insurance coverage |  |  |
| No | 56.2 | 54.8-57.6 |
| Yes | 66.8 | 64.7-68.9 |

The treatment of hypertension based on the socioeconomic characteristics is given in Table . We found that 80.2% (78.9-81.4) of hypertensive individuals were receiving treatment. By increase in age, the rate of treatment increases. Females had higher rate of treatment than males. An increase in the years of schooling decreases the rate of treatment.

The control of hypertension based on the socioeconomic characteristics is given in Table 4. The control rate of HTN was 39.1% (37.4-40.7). Control decreased from 62.9% among the 25-34-year-old age group to 34.5% among the 65-74-year-old age group (Table 4). Subsequently, it increased among individuals ≥75 years.

Table 3. Percentage of hypertension treatment based on socioeconomic characteristics of study participants

|  | JNC8 |  |
| --- | --- | --- |
|  | %^†^ | 95% CI |
| Overall | 80.2 | 78.9-81.4 |
| Age groups (years old) |  |  |
| 25-34 | 44.1 | 34.7-53.9 |
| 35-44 | 61.2 | 55.9-66.2 |
| 45-54 | 75.4 | 72.4-78.3 |
| 55-64 | 81.4 | 79.2-83.5 |
| 65-74 | 86.6 | 84.3-88.6 |
| ≥75 | 88.2 | 85.6-90.3 |
| Gender |  |  |
| Male | 78.2 | 76.2-80.1 |
| Female | 81.3 | 79.8-82.8 |
| Area of residence |  |  |
| Urban | 80.8 | 79.3-82.2 |
| Rural | 78.7 | 76.2-81.0 |
| Wealth status |  |  |
| Poorest | 79.7 | 76.8-82.3 |
| Poor | 82.6 | 80.0-84.8 |
| Average | 78.5 | 75.5-81.1 |
| Rich | 78.6 | 75.5-81.3 |
| Richest | 79.7 | 76.5-82.6 |
| Years of schooling |  |  |
| No schooling | 83.7 | 81.7-85.4 |
| 1-6 years | 79.8 | 77.5-81.8 |
| 7-12 years | 75.5 | 72.6-78.2 |
| >12years | 79.0 | 74.9-82.5 |
| Complementary health insurance coverage |  |  |
| No | 78.3 | 76.7-79.9 |
| Yes | 84.2 | 82.2-86.1 |

Table 4. Percentage hypertension control based on socioeconomic characteristics of study participants

| Control (%) | %^†^ | 95% CI |
| --- | --- | --- |
| Overall | 39.1 | 37.4-40.7 |
| Age groups (years old) |  |  |
| 25-34 | 62.9 | 47.9-75.8 |
| 35-44 | 51.4 | 44.4-58.4 |
| 45-54 | 40.0 | 36.3-43.8 |
| 55-64 | 38.9 | 35.9-42.0 |
| 65-74 | 34.5 | 31.4-37.7 |
| ≥75 | 39.0 | 35.4-42.8 |
| Gender |  |  |
| Male | 40.9 | 38.2-43.6 |
| Female | 38.0 | 36.0-40.1 |
| Area of residence |  |  |
| Urban | 40.7 | 38.8-42.7 |
| Rural | 35.0 | 32.0-38.2 |
| Wealth **status** |  |  |
| Poorest | 37.5 | 33.8-41.3 |
| Poor | 32.7 | 29.6-35.9 |
| Average | 38.1 | 34.3-41.9 |
| Rich | 43.8 | 39.9-47.9 |
| Richest | 45.3 | 41.2-49.5 |
| Years of schooling |  |  |
| No schooling | 33.0 | 30.6-35.6 |
| 1-6 years | 41.2 | 38.2-44.3 |
| 7-12 years | 45.5 | 41.9-49.1 |
| >12 years | 42.2 | 37.1-47.5 |
| Complementary health insurance coverage |  |  |
| No | 37.3 | 35.3-39.4 |
| Yes | 43.1 | 40.2-46.1 |

## Comparison of concentration index at the provincial level

The comparison of concentration index of hypertension prevalence between provinces is given in Table 5. Provinces are ranked from the largest to the smallest C that disfavors the poorest groups. The largest concentration index belongs to Gillan province with -0.205. In the second rank is Yazd (C=-0.171) and in the third rank is Chahar Mahl (C=-0.166). Out of 30 provinces, 14 provinces have inequality of hypertension prevalence that was statistically significant. The null hypothesis that there is no difference between provinces regarding C of prevalence was strongly reject (F=3.829, p<001).

Table 5. Comparison of concentration index for hypertension prevalence between provinces*

|  | Province | No. of observation | Concentration index | Robust std. error | p-value |
| --- | --- | --- | --- | --- | --- |
| 1 | Gilan | 886 | -0.205 | 0.037 | 0.000 |
| 2 | Yazd | 628 | -0.171 | 0.042 | 0.000 |
| 3 | Charhar Mahal Bakhtiyari | 572 | -0.166 | 0.042 | 0.000 |
| 4 | Isfahan | 1721 | -0.152 | 0.025 | 0.000 |
| 5 | Mazandaran | 1042 | -0.150 | 0.034 | 0.000 |
| 6 | Markazi | 498 | -0.146 | 0.048 | 0.003 |
| 7 | Zanjan | 680 | -0.145 | 0.036 | 0.000 |
| 8 | Gazvin | 402 | -0.136 | 0.052 | 0.009 |
| 9 | East Azarbaijan | 1215 | -0.117 | 0.029 | 0.000 |
| 10 | Khorasan North | 566 | -0.117 | 0.045 | 0.010 |
| 11 | Hamedan | 598 | -0.099 | 0.040 | 0.014 |
| 12 | Fars | 1563 | -0.083 | 0.025 | 0.001 |
| 13 | Ardabil | 402 | -0.079 | 0.055 | 0.151 |
| 14 | Alborz | 900 | -0.065 | 0.035 | 0.063 |
| 15 | Khorasan razavi | 1943 | -0.061 | 0.023 | 0.008 |
| 16 | Lorestan | 549 | -0.055 | 0.046 | 0.235 |
| 17 | Tehran | 3947 | -0.053 | 0.016 | 0.001 |
| 18 | Ilam | 385 | -0.050 | 0.054 | 0.353 |
| 19 | Golestan | 546 | -0.049 | 0.048 | 0.307 |
| 20 | Kerman | 977 | -0.043 | 0.031 | 0.163 |
| 21 | West Azarbaijan | 1021 | -0.024 | 0.033 | 0.461 |
| 22 | Kurdestan | 503 | -0.021 | 0.050 | 0.680 |
| 23 | Kohkiloye | 421 | -0.016 | 0.052 | 0.763 |
| 24 | Khorasan South | 488 | -0.015 | 0.045 | 0.735 |
| 25 | Semnan | 442 | -0.011 | 0.058 | 0.849 |
| 26 | khuzestan | 1370 | 0.012 | 0.029 | 0.675 |
| 27 | Hormozgan | 493 | 0.035 | 0.032 | 0.267 |
| 28 | Kermanshah | 672 | 0.037 | 0.041 | 0.364 |
| 29 | Sistan | 665 | 0.038 | 0.022 | 0.091 |
| 30 | Boushehr | 340 | 0.058 | 0.061 | 0.342 |

*Note: comparison between provinces; F test=3.829, p<.001.

The comparison of concentration index of hypertension awareness between provinces is given . Provinces are ranked from the largest positive C to the smallest ones that disfavors the poorest groups. The largest concentration index belonged to Lorestan and Hormozgan provinces with 0.231 and 0.123, respectively. The concentration index in these provinces was significant indicating that there were significant differences between richest and poorest groups with regard to the awareness of hypertension and that the inequality disadvantaged poorest groups. On the other hand, in four provinces Gillan, Ardabil, Illam, and Semnan there was significant wealth-related inequality that disfavored the richest wealth groups as C is negative in these provinces. The difference between provinces in the awareness of hypertension was statistically significant (F=1.610, p<0.05).

Table 6. Comparison of concentration index for hypertension awareness between provinces*

|  | Province | No. of observation | Concentration index | Robust std. error | p-value |
| --- | --- | --- | --- | --- | --- |
| 1 | Lorestan | 199 | 0.231 | 0.076 | 0.003 |
| 2 | Hormozgan | 156 | 0.123 | 0.061 | 0.047 |
| 3 | Kohkiloye | 112 | 0.107 | 0.097 | 0.272 |
| 4 | West Azarbaijan | 335 | 0.106 | 0.060 | 0.076 |
| 5 | Sistan | 160 | 0.073 | 0.047 | 0.126 |
| 6 | Khorasan South | 146 | 0.064 | 0.091 | 0.482 |
| 7 | khuzestan | 418 | 0.044 | 0.057 | 0.439 |
| 8 | Charhar Mahal Bakhtiyari | 184 | 0.042 | 0.078 | 0.589 |
| 9 | Kerman | 283 | 0.028 | 0.061 | 0.640 |
| 10 | Zanjan | 170 | -0.001 | 0.086 | 0.989 |
| 11 | Tehran | 1091 | -0.015 | 0.034 | 0.665 |
| 12 | Hamedan | 145 | -0.019 | 0.092 | 0.839 |
| 13 | Khorasan North | 197 | -0.024 | 0.079 | 0.761 |
| 14 | Fars | 407 | -0.026 | 0.054 | 0.622 |
| 15 | Alborz | 279 | -0.035 | 0.067 | 0.601 |
| 16 | Mazandaran | 354 | -0.041 | 0.061 | 0.497 |
| 17 | Kermanshah | 201 | -0.046 | 0.081 | 0.569 |
| 18 | East Azarbaijan | 396 | -0.047 | 0.056 | 0.401 |
| 19 | Gazvin | 113 | -0.049 | 0.107 | 0.651 |
| 20 | Markazi | 170 | -0.050 | 0.088 | 0.573 |
| 21 | Golestan | 177 | -0.054 | 0.092 | 0.557 |
| 22 | Isfahan | 507 | -0.055 | 0.048 | 0.249 |
| 23 | Khorasan razavi | 565 | -0.058 | 0.046 | 0.209 |
| 24 | Kordestan | 182 | -0.095 | 0.087 | 0.276 |
| 25 | Yazd | 191 | -0.133 | 0.072 | 0.068 |
| 26 | Gilan | 311 | -0.143 | 0.064 | 0.025 |
| 27 | Ardabil | 155 | -0.177 | 0.086 | 0.041 |
| 28 | Boushehr | 80 | -0.189 | 0.125 | 0.133 |
| 29 | Ilam | 121 | -0.204 | 0.098 | 0.039 |
| 30 | Semnan | 176 | -0.247 | 0.081 | 0.003 |

*Note: comparison between provinces; F test=1.610, p<.05.

The comparison of C of hypertension treatment between provinces is given in Table . The largest positive C belongs to Illam province with 0.214 (p<0.05). This finding shows that wealth inequality in hypertension treatment in this province was in disadvantage of the poorest group. In contrast, Tehran, Azarbaijan Sharghi, Hamedan, and North Khorasan had negative and significant C indicating that treatment was at the disadvantage of the richest wealth groups. There was significant difference between provinces regarding treatment rate for patients with hypertension (F=1.58, p<0.05)

Table 7. Comparison of concentration index for hypertension treatment between provinces*

|  | Province | No. of observation | Concentration index | Robust std. error | p-value |
| --- | --- | --- | --- | --- | --- |
| 1 | Ilam | 69 | 0.214 | 0.103 | 0.041 |
| 2 | Hormozgan | 65 | 0.142 | 0.087 | 0.106 |
| 3 | Ardabil | 96 | 0.127 | 0.068 | 0.063 |
| 4 | Kermanshah | 107 | 0.107 | 0.081 | 0.189 |
| 5 | Kerman | 191 | 0.080 | 0.054 | 0.140 |
| 6 | Golestan | 106 | 0.065 | 0.072 | 0.368 |
| 7 | Sistan | 105 | 0.060 | 0.037 | 0.109 |
| 8 | Gilan | 173 | 0.053 | 0.068 | 0.443 |
| 9 | Zanjan | 91 | 0.045 | 0.102 | 0.660 |
| 10 | khuzestan | 230 | 0.030 | 0.057 | 0.599 |
| 11 | Markazi | 104 | 0.025 | 0.078 | 0.753 |
| 12 | Fars | 266 | 0.021 | 0.058 | 0.722 |
| 13 | Mazandaran | 209 | 0.012 | 0.072 | 0.871 |
| 14 | East Azarbaijan | 231 | -0.008 | 0.068 | 0.908 |
| 15 | Alborz | 162 | -0.009 | 0.066 | 0.889 |
| 16 | Semnan | 104 | -0.029 | 0.072 | 0.691 |
| 17 | Isfahan | 333 | -0.029 | 0.049 | 0.549 |
| 18 | Lorestan | 114 | -0.034 | 0.092 | 0.710 |
| 19 | Kordestan | 101 | -0.044 | 0.095 | 0.639 |
| 20 | Charhar Mahal Bakhtiyari | 110 | -0.047 | 0.060 | 0.431 |
| 21 | Gazvin | 63 | -0.055 | 0.138 | 0.690 |
| 22 | Khorasan South | 76 | -0.070 | 0.092 | 0.448 |
| 23 | Tehran | 623 | -0.073 | 0.033 | 0.029 |
| 24 | Khorasan razavi | 357 | -0.073 | 0.051 | 0.151 |
| 25 | Yazd | 127 | -0.079 | 0.085 | 0.359 |
| 26 | Boushehr | 37 | -0.084 | 0.162 | 0.605 |
| 27 | Kohkiloye | 78 | -0.109 | 0.113 | 0.338 |
| 28 | West Azarbaijan | 202 | -0.129 | 0.064 | 0.046 |
| 29 | Hamedan | 88 | -0.170 | 0.081 | 0.040 |
| 30 | Khorasan North | 127 | -0.174 | 0.081 | 0.033 |

*Note: comparison between provinces; F test=1.588, p<.05.

The comparison of C of hypertension control between provinces is given in **Error! Reference source not found.**. Top rank provinces with the largest positive concentration index of hypertension control were Lorestan, West Azarbaijan, Kurdestan, and Ardabil with 0.342, 0.274, 0.272, and 0.248 (p value<0.05). In these provinces, wealth related inequality in the control of hypertension disadvantaged the poorest group. Furthermore, Khuzestan and Isfahan had significant inequality that was also at the disadvantage of poorest groups. While there are provinces such as Semnan, Khorsan Razavi where C was negative indicating that wealth-related inequality was at the disadvantage of richest group, the difference between poorest and richest groups was nonsignificant. The overall difference between the provinces in terms of hypertension control was also nonsignificant (F=1.292, p=0.136).

Table 8. Comparison of concentration index for hypertension control between provinces*

|  | Province | No. of observation | Concentration index | Robust std. error | p-value |
| --- | --- | --- | --- | --- | --- |
| 1 | Lorestan | 87 | 0.342 | 0.107 | 0.002 |
| 2 | West Azarbaijan | 154 | 0.274 | 0.086 | 0.002 |
| 3 | Kordestan | 79 | 0.272 | 0.116 | 0.022 |
| 4 | Ardabil | 87 | 0.248 | 0.116 | 0.035 |
| 5 | Hamedan | 71 | 0.238 | 0.126 | 0.063 |
| 6 | khuzestan | 182 | 0.211 | 0.076 | 0.006 |
| 7 | Yazd | 107 | 0.194 | 0.108 | 0.075 |
| 8 | Zanjan | 65 | 0.177 | 0.135 | 0.196 |
| 9 | Isfahan | 266 | 0.164 | 0.067 | 0.016 |
| 10 | East Azarbaijan | 168 | 0.156 | 0.083 | 0.062 |
| 11 | Alborz | 137 | 0.140 | 0.088 | 0.115 |
| 12 | Fars | 198 | 0.120 | 0.080 | 0.136 |
| 13 | Kohkiloye | 55 | 0.117 | 0.153 | 0.446 |
| 14 | Ilam | 56 | 0.117 | 0.151 | 0.445 |
| 15 | Mazandaran | 162 | 0.106 | 0.085 | 0.214 |
| 16 | Charhar Mahal Bakhtiyari | 99 | 0.098 | 0.099 | 0.327 |
| 17 | Kerman | 161 | 0.091 | 0.086 | 0.289 |
| 18 | Golestan | 86 | 0.055 | 0.135 | 0.685 |
| 19 | Sistan | 91 | 0.048 | 0.083 | 0.569 |
| 20 | Markazi | 89 | 0.030 | 0.110 | 0.787 |
| 21 | Khorasan North | 100 | 0.017 | 0.112 | 0.880 |
| 22 | Tehran | 518 | 0.014 | 0.049 | 0.772 |
| 23 | Gilan | 141 | 0.007 | 0.109 | 0.951 |
| 24 | Semnan | 88 | -0.002 | 0.148 | 0.988 |
| 25 | Khorasan razavi | 275 | -0.034 | 0.068 | 0.613 |
| 26 | Kermanshah | 81 | -0.078 | 0.109 | 0.477 |
| 27 | Hormozgan | 48 | -0.131 | 0.138 | 0.345 |
| 28 | Gazvin | 43 | -0.132 | 0.187 | 0.486 |
| 29 | Khorasan South | 62 | -0.176 | 0.131 | 0.184 |
| 30 | Boushehr | 30 | -0.210 | 0.186 | 0.267 |

*Note: comparison between provinces; F test=1.292, p=0.136.
